# Synergistic effects of cognitive frailty and comorbidities on disability: a community-based longitudinal study

**DOI:** 10.1186/s12877-024-05057-3

**Published:** 2024-05-23

**Authors:** Nurul Fatin Malek Rivan, Resshaya Roobini Murukesu, Suzana Shahar, Nor Fadilah Rajab, Ponnusamy Subramaniam, Theng Choon Ooi, Mohd Zul Amin Kamaruddin, Devinder Kaur Ajit Singh

**Affiliations:** 1 Nutritional Sciences Programme and Centre for Healthy Ageing and Wellness (H-CARE), Faculty of Health Sciences, Universiti Kebangsaan Malaysia, Jalan Raja Muda Abdul Aziz, Kuala Lumpur, 50300 Malaysia; 2 Physiotherapy Programme & Centre for Healthy Ageing and Wellness (H-CARE), Universiti Kebangsaan Malaysia, Jalan Raja Muda Abdul Aziz, Kuala Lumpur, 50300 Malaysia; 3 Dietetics Programme and Centre for Healthy Ageing and Wellness (H-CARE), Faculty of Health Sciences, Universiti Kebangsaan Malaysia, Jalan Raja Muda Abdul Aziz, Kuala Lumpur, 50300 Malaysia; 4 Biomedical Science Programme, Centre for Healthy Ageing and Wellness (H-CARE), Faculty of Health Sciences, Universiti Kebangsaan Malaysia, Jalan Raja Muda Abdul Aziz, Kuala Lumpur, 50300 Malaysia; 5 Health Psychology Programme and Centre of Rehabilitation Science, Faculty of Health Sciences, Universiti Kebangsaan Malaysia, Jalan Raja Muda Abdul Aziz, Kuala Lumpur, 50300 Malaysia; 6 Centre for Healthy Ageing and Wellness (H-CARE), Faculty of Health Sciences, Universiti Kebangsaan Malaysia, Jalan Raja Muda Abdul Aziz, Kuala Lumpur, 50300 Malaysia; 7 TUM School of Medicine & Health, Department of Health and Sport Sciences, Technical University of Munich, Munich, Germany

**Keywords:** Cognitive frailty, Morbidity, Synergism, Disability, Older adults

## Abstract

**Objective:**

In this study, we aimed to assess the synergistic effects of cognitive frailty (CF) and comorbidity on disability among older adults.

**Methods:**

Out of the 1318 participants from the Malaysian Towards Useful Aging (TUA) study, only 400 were included in the five-year follow-up analysis. A comprehensive interview-based questionnaire covering socio-demographic information, health status, biochemical indices, cognitive and physical function, and psychosocial factors was administered. Binary logistic regression analysis was employed to estimate the independent and combined odd ratios (ORs). Measures such as the relative excess risk due to interaction (RERI), the attributable proportion of risk due to the interaction, and the synergy index were used to assess the interaction between CF and comorbidity.

**Results:**

Participants with CF (24.1%) were more likely to report disability compared to those without CF (10.3%). Synergistic effects impacting disability were observed between CF and osteoarthritis (OA) (OR: 6.675, 95% CI: 1.057–42.158; RERI: 1.501, 95% CI: 1.400–1.570), CF and heart diseases (HD) (OR: 3.480, 95% CI: 1.378–8.786; RERI: 0.875, 95% CI: 0.831–0.919), CF and depressive symptoms (OR: 3.443, 95% CI: 1.065–11.126; RERI: 0.806, 95% CI: 0.753–0.859), and between CF and diabetes mellitus (DM) (OR: 2.904, 95% Confidence Interval (CI): 1.487–5.671; RERI: 0.607, 95% CI: 0.577–0.637).

**Conclusion:**

These findings highlight the synergism between the co-existence of CF and comorbidity, including OA, HD, DM, and depressive symptoms, on disability in older adults. Screening, assessing, and managing comorbidities, especially OA, HD, DM and depressive symptoms, when managing older adults with CF are crucial for reducing the risk of or preventing the development of disability.

## Introduction

Approximately 15% of the global population is estimated to have some form of disability due to the rapid rise in the global aging population and a parallel increase in the prevalence of chronic health conditions [1]. With the progressive surge in longevity and lifespan, disability is steadily becoming an integral factor of disease burden worldwide. Several chronic diseases, including ischemic heart disease, stroke, diabetes, and dementia in mid-life or late life, have been identified as causes of disability-adjusted life years (DALYs), which is the sum of years lost due to premature mortality [2]. Functional disability in late life appears to be linked with gradual age-related deterioration and the coexistence of multiple diseases [3, 4]. Consequently, this strongly predicts future needs for assisted living and long-term nursing care, which heavily burden the healthcare system in addition to economic and personal burdens [2]. Disability risk factors include gender, age, socioeconomic status, lifestyle, and chronic diseases [5, 6]. This phenomenon is also a known adverse outcome of both frailty and cognitive impairment in older adults [7, 8].

Notably, frailty and cognitive impairment were often viewed as two independent concepts in previous studies until cognitive frailty (CF) was introduced by the consensus group of the International Academy on Nutrition and Aging (IANA) and the International Association of Gerontology and Geriatrics (IAGG) [9]. The simultaneous coexistence of physical frailty and cognitive impairment, or CF, seems to entail a greater risk of all-cause mortality and adverse health outcomes than their respective effects alone, as reported in both community-based and population-based studies [10–12]. The higher prevalence and incidence of CF, specifically among community-dwelling Malaysian older adults (39.6%; 7.1 per 100 person-years), has become a worrisome issue as those with CF are predicted to develop disability incidence by fivefold as compared to frailty and mild cognitive impairment on its own, based on five years cohort study [13–15].

Both physical frailty and cognitive impairment have been identified as risk factors for physical disability [8, 15], where these may act independently or, more often, in synergistic combinations. Individuals with CF are more likely to experience difficulties in performing activities of daily living (ADLs) and instrumental activities of daily living (IADLs) by two- to fivefold, leading to functional impairment and dependence on others for care [16]. Chronic low-grade inflammation, often observed in individuals with CF, can impair the regeneration of muscle tissue following injury and exacerbate muscle mass loss and functional decline, limiting an individual’s ability to perform activities of daily living and subsequent disability incidence [15, 17].

Furthermore, the presence of chronic diseases in older adults with CF further exacerbates the decline of physiological reserve function in multiple systems, thereby increasing the risk of adverse health outcomes [18]. Different chronic disease profiles or comorbidity patterns, defined as the co-occurrence of two or more chronic conditions in an individual with CF, may impersonate clinically different etiologic pathways to the occurrence of disability in late life. The most common conditions among older adults with CF are diabetes mellitus, hypertension, depression, and cardiovascular disease [19–21]. The simultaneous presence of two or more chronic illnesses that include high depressive symptoms is associated with high rates of prospective ADL-IADL disability, potentially due to polypharmacy adverse reactions [22, 23]. Different comorbidity patterns may influence the pathways to disability in late life, accentuating the need for comprehensive assessment and management strategies tailored to the specific combination of chronic conditions in individuals with CF.

However, to date, the joint effect of CF and comorbidity on disability incidence has not been investigated. Seeing as Malaysia is on the cusp of transforming into an aging nation, assessing older adults with CF and comorbidity in this population may enable the identification of older adults who are more at risk for disability earlier to implement a more effective intervention. We hypothesised that the presence of comorbidity in older adults with CF has differential effects on the incidence of disability and may exceed the sum of their effects alone. Thus, the aim in this prospective cohort analysis was to evaluate the synergistic effects of CF and comorbidity on the incidence of disability among Malaysian older adults aged 60 years and above.

## Methods

### Study design and participants

This is a follow-up study of the Long-Term Research Grant Scheme – Towards Useful Aging (LRGS TUA) cohort [24] at five years endpoint. LRGS-TUA is a prospective cohort study of community-dwelling older adults aged 60 years and above in Malaysia. They were recruited through a multi-stage random sampling procedure from two states with the highest prevalence of older adults, namely Selangor and Perak (representing central and northern regions of Malaysia). The study employed a three-stage sampling process that involved selecting primary sampling units (PSUs) (i.e., states), followed by the selection of secondary sampling units (SSUs) (i.e., a random census circle within the state), and finally, the selection of tertiary sampling units (TSUs) (i.e., living quarters within the census circle). Probability sampling weights were performed at each stage to ensure that the study sample accurately represents the target population.

This study involved two data collection phases: baseline (collected in 2013) and after five years of follow-up (collected in 2018) prior to obtaining insights into the trajectory of participants’ disability status over the study period. From this process, a total of 815 participants who had complete physical frailty and cognitive statuses data at baseline were analyzed. At baseline, older adults diagnosed with dementia, known psychiatric conditions, severe vision, speech, and hearing problems, as well as those who were non-ambulant, were excluded to ensure the reliability of the data collected. The selected participants should be able to converse in either Malay, English, Chinese or Tamil languages and agree to sign the consent form to participate in this study.

A total of 400 participants (49.1%) were successfully followed up at five years. Whilst 4.8% had passed away, and 46.1% of the participants refused to participate or failed to be contacted. A prior study reported a fairly high drop-out rate among the participants who were older in age and lived alone, which restricted their participation, possibly due to a lack of support for acquiring care and transportation difficulties [14]. Furthermore, potential variations attributed to seasonal and regional factors were considered during the follow-up assessment, as the data collection was conducted within the same month as the baseline, spanning from August to April. The study has been approved by the Medical Research and Ethics Committee of the Universiti Kebangsaan Malaysia (UKM1.21.3/244/NN-2018-145). Before participation, each participant provided informed, written consent and was given the assurance of anonymity.

### Data collection

Trained enumerators conducted structured interviews with the participants, and measurements were taken for various parameters at nearby community centers. The questionnaire used was to gather data on socio-demographic information, health status, neuropsychological and psychosocial functions, lifestyle, blood pressure, anthropometry, biochemical indices, and functional status. The baseline assessments were repeated five years later at follow-up. All procedures were conducted in compliance with relevant guidelines and regulations, as detailed in the study by Shahar et al. [24]. Participants were given rest breaks during the tests, and monetary incentives were provided for completing the assessments.

### Operationalisation of cognitive frailty (CF)

As described in our prior report [13, 14], the classification of CF was based on the concurrent existence of physical pre-frailty/frailty and subjective cognitive decline/mild cognitive impairment (MCI) at baseline. Participants were then divided into two groups, CF and Non-CF groups.

### Physical frailty

Physical frailty was assessed using Fried et al. [25] criteria and the cut-off points outlined in the Cardiovascular Health Study (CHS). The assessment’s variables were categorized as follows: (1) unintentional weight loss of more than 5 kg in the previous years; (2) self-reported exhaustion or tiredness based on two items of the Centre of Epidemiologic Studies Depression scale (CES-D): (a) ‘I felt that everything I did was an effort’ and (b) ‘I could not get going’; (the question was how often in the last week did you feel like this (rare = 0, some or little time (1–2 days) = 1, moderate (3–4 days) = 2, and most of the times = 3); 3) low physical activity assessed using the Physical Activity Scale for Elderly (PASE) [26]; 4) weakness measured using dominant hand grip strength (digital hand dynamometer; Jamar® Plus+, Patternson Medical, IL, USA); and 5) slowness, measured using the five-metre gait speed test. Participants who meet three out of the five criteria are typically classified as frail, while those who meet 1 to 2 of these criteria are generally categorized as pre-frail.

### Mild cognitive impairment (MCI)

Mild cognitive impairment (MCI) is a clinical condition characterized by cognitive decline that is greater than expected for an individual’s age and educational background but does not significantly impair daily functioning or independence in activities of daily living. Participants were classified as having MCI if they exhibited subjective memory complaint (reported by the participants based on item ten of the 15-item Geriatric Depression Scale or their caregivers) along with objective memory impairment (at least 1.5 standard deviations below the mean for Rey Auditory Verbal Learning Test, RAVLT), but with minimal to no functional limitations in basic activities of daily living (at least 1.5 standard deviations below the mean). The subjective memory complaints are the self-reported cognitive symptoms, particularly memory complaints, for those who perceive a decline in their cognitive abilities. Additionally, they were required to maintain global cognitive function (indicated by a Malay version of Mini-Mental State Examination, M-MMSE score of *≥* 19) and not diagnosed with dementia by a physician at baseline. Those participants with M-MMSE < 19 were excluded from the study as it is generally considered indicative of moderate to severe cognitive impairment. These criteria were established based on Petersen et al. [27] and Lee et al. [28]. Participants who did not meet these criteria were categorized as normal.

### Comorbidities

Self-reported data was employed to determine the presence of comorbidity, which is defined as the co-occurrence of two disorders within a single individual. Self-reported information on certain diseases (hypertension, hypercholesterolemia, diabetes mellitus, osteoarthritis, heart disease, chronic kidney disease, cancer) diagnosed by doctors in the prior years was recorded. Self-report of disease diagnoses has been frequently used in epidemiology studies and reported to be valid [29]. Meanwhile, depressive symptoms were assessed using the geriatric depression scale (GDS-15), where those with a score of five and above were categorized as depressed [30].

### Outcome measure

#### Disability

The data on disability was measured at five-years follow up. To assess restrictions in daily activities and social participation resulting from health problems in the past month, The World Health Organization Disability Assessment Schedule (WHODAS, version 2.0), a 12-item questionnaire with a five-point Likert scale (ranging from 0 for none to 4 for very severe) was used [31]. WHODAS was used to evaluate disability, which covers six main areas, including self-care, participation, cognition, mobility, getting along, and life activities. The degree of disability was classified as follows: 0 (no disability), 1–4 (mild disability), 5–9 (moderate disability), and 10–48 or more (severe disability). In this study, participants were divided into two groups based on their disability status: those with severe disability and those without disability (all others).

### Potential confounding factors

Multiple factors assessed at baseline were considered confounding factors that may affect the occurrence of disability over an extended period of time. These elements were previously addressed in detail [24], including:

### Socio-demographic information and medical history

The information collected included the participant’s age, gender, ethnicity, level of education, household income, living situation (living alone or with others), and whether or not they smoked.

### Nutritional status, body composition, and blood pressure

The study included various anthropometric measurements such as height, weight, waist circumference, mid-upper arm circumference (MUAC), and calf circumference. Body mass index (BMI) was calculated by dividing the body weight in kilograms by the square of the standing height in meters. Additionally, body composition was assessed using the Bio-electrical Impedance Analysis InBody S10 (Biospace, Seoul, Korea). Furthermore, systolic and diastolic blood pressure were recorded using a calibrated digital automatic blood pressure monitor (OMRON, Kyoto Japan).

### Laboratory analysis

A certified phlebotomist took 20 ml of fasting peripheral venous blood using a butterfly syringe for the biochemical analysis, which included fasting blood sugar (FBS), Haemoglobin A1c (HbA1c), total cholesterol (TC), high-density lipoprotein (HDL), low-density lipoprotein (LDL), and albumin (ALB).

### Psychosocial and functional assessments

The assessment of social support was conducted using the Medical Outcome Study Social Support Survey (MOSS) [32], while the functional status was evaluated using the Instrumental Activities of Daily Living (IADL) scale [33].

### Statistical analysis

Descriptive statistics were utilized to identify differences in participant characteristics between the CF and Non-CF groups at baseline, in order to determine any potential confounding factors. Categorical variables were compared using the Chi-Square test (χ2), while continuous variables were compared using the independent t-test. The factors reported were based on the baseline data, rather than follow-up data. The findings were presented as n (%) for categorical data and mean ± standard deviation for normally distributed continuous data. These statistical analyses were conducted using SPSS version 23.0 (Licensed materials - Property of SPSS Incorporation an IBM Company Copyright 1989 and 2010 SPSS, Chicago, United States).

A binary logistic regression model (BLR) was conducted to estimate the independent effects of each health condition on the incidence of disability and adjusted for the confounding factors. Then, the hypothesis of the combined effect of CF and comorbidity on the disability incidence was further tested using BLR. To assess multicollinearity among predictor variables, tolerance and variance inflation factor (VIF) values were also computed. Tolerance values below 0.2 and VIF values exceeding 5 were considered indicative of potential multicollinearity.

### Relative excess risk due to interaction (rERI)

Each model used three dummy (indicator) variables to represent the three comparisons made with the reference category of either having CF or having a comorbidity, or having both (RR_11_); CF without the comorbidity (RR_10_); and the comorbidity without CF (RR_01_). The three measures of synergism were calculated based on Rothman’s definitions [34]. The RERI, which stands for relative excess risk due to interaction, represents the additional risk that arises from the interaction between CF and comorbidity in individuals who have both conditions. This extra risk is calculated by subtracting the sum of the individual effects of each condition from the combined effect. This is calculated by the following equation: RERI_RR_ = RR_11_ - RR_10_ - RR_01_ + 1. The AP, or attributable proportion due to interaction, refers to the portion of the combined RR (RR_11_) that results specifically from the interaction between the two conditions. While, the SI, or synergy index, is the ratio of the combined effect to the sum of the individual effects of the two conditions [34]. Additionally, the descriptive command was used to calculate the 95% confidence interval based on the standard error of the mean for RERI, AP, and SI.

The AP and the SI provide information on the magnitude of the impacts, while the RERI provides the direction of any synergistic effects [35]. The RERI is seen as the primary indicator that synergistic effects exist. A RERI of 0 denotes that the two conditions do not interact and act independently. When the RERI value is negative, it indicates that the two conditions are interacting in such a way that they lower the risk of the outcome. Conversely, a positive RERI value suggests that the interaction between the two conditions is increasing the risk of the outcome. The synergistic effect can account for 0–100% of the total RR, depending on the range of the AP from 0.0 to 1.0. If the SI is larger than 1.0, the combined impact of the conditions is greater than the sum of the individual effects. At the extreme, RERI and SI would approach infinity, and the AP would approach 1.0 when two conditions have no influence independently but a measurable effect when combined [34].

## Results

### Characteristics of the participants

Of the 815 participants enrolled at baseline, only 400 were included in the follow-up analysis. Table 1 compares the demographic characteristics of participants with CF (*N* = 158) and without CF, known as non-CF (*N* = 242). Participants with CF were older (mean: 68.61 ± 5.52 years old), mostly female (58.9%) and of non-Malay ethnicities (65.8%). CF participants had significantly lower years of education (5.41 ± 4.33 years) and household income (RM1340.85 ± 1660.38/USD304.74 ± 377.36) as compared to the non-CF participants (RM441.53 ± 2808.38/USD441.53 ± 638.27) (*p* < 0.05). Those with CF also had higher body fat percentage (39.98 ± 9.82% vs. 37.80 ± 10.22%), and lower skeletal muscle mass (19.80 ± 4.58 kg vs. 20.98 ± 5.00 kg), than the non-CF participants (*p* < 0.05). Additionally, CF participants were observed to have significantly lower social support (33.89 ± 15.58 vs. 38.92 ± 14.67), and poorer functional status (12.99 ± 1.68 vs. 13.26 ± 1.45), as compared to non-CF participants (*p* < 0.05).

**Table 1:** The baseline characteristics of the participants according to the groups of Non-CF and CF at baseline [presented as mean ± standard deviation (sd) or n (%)]

	Total (*N* = 400)	Non-CF (*N* = 242)	CF (*N* = 158)	*p*-value
Age (years)	67.63 ± 5.26	67.00 ± 4.99	68.61 ± 5.52	0.003*
Gender				
Male	180 (45.0)	115 (47.5)	65 (41.1)	0.219
Female	220 (55.0)	127 (52.5)	93 (58.9)	
Ethnicity				
Malay	162 (40.5)	108 (44.6)	54 (34.2)	0.048*
Chinese & Indian	238 (59.5)	134 (55.4)	104 (65.8)	
Years of education (years)	6.36 ± 4.56	6.99 ± 4.61	5.41 ± 4.33	0.001*
Household income (RM)	1704.43 ± 2435.25	1942.74 ± 2808.38	1340.85 ± 1660.38	0.008*
Living arrangement				
Living with others	361 (90.3)	224 (92.6)	137 (86.7)	0.059
Living alone	39 (9.7)	18 (7.4)	21 (13.3)	
Smoking status				
Non-smoker	320 (80.0)	197 (81.4)	123 (77.8)	0.443
Smoker	80 (20.0)	45 (18.6)	35 (22.2)	
Blood pressure				
SBP (mmHg)	138.15 ± 18.77	138.50 ± 19.39	137.61 ± 17.85	0.648
DBP (mmHg)	76.42 ± 12.52	77.17 ± 12.29	75.32 ± 12.81	0.155
Nutritional assessments				
BMI (kg/m^2^)	25.50 ± 4.04	25.34 ± 3.96	25.75 ± 4.17	0.325
CC (cm)	34.38 ± 3.48	34.44 ± 3.45	34.30 ± 3.53	0.706
WC (cm)	89.32 ± 10.40	89.00 ± 10.31	89.80 ± 10.55	0.452
MUAC (cm)	29.03 ± 3.15	29.06 ± 3.12	28.99 ± 3.20	0.830
Body composition				
% Body fat	38.66 ± 10.11	37.80 ± 10.22	39.98 ± 9.82	0.036*
SMM (kg)	13.15 ± 1.55	20.98 ± 5.00	19.80 ± 4.58	0.018*
Laboratory analysis				
FBS (mmol/l)	5.94 ± 2.01	6.11 ± 2.36	5.68 ± 1.26	0.050
TC (mmol/l)	5.18 ± 1.01	5.18 ± 1.02	5.19 ± 0.99	0.969
HDL (mmol/l)	1.46 ± 0.37	1.44 ± 0.39	1.48 ± 0.35	0.300
LDL (mmol/l)	3.07 ± 0.91	3.09 ± 0.95	3.04 ± 0.86	0.576
Triglyceride (mmol/l)	1.45 ± 0.74	1.44 ± 0.76	1.47 ± 0.70	0.706
Albumin (g/l)	42.99 ± 2.70	43.03 ± 2.94	42.92 ± 2.31	0.715
Psychosocial and functional status				
MOSS	36.91 ± 15.22	38.92 ± 14.67	33.89 ± 15.58	0.001*
IADL	13.15 ± 1.55	13.26 ± 1.45	12.99 ± 1.68	0.096

*Significant at *p* < 0.05 using one-way ANOVA test. Notes: CF = cognitive frailty; BMI = Body Mass Index; CC = Calf circumference; WC = Waist circumference; MUAC = Mid-upper arm circumferences; SBP = Systolic diastolic pressure; DBP = Diastolic blood pressure; SMM = Skeletal muscle mass; FBS = Fasting Blood Sugar; TC = Total cholesterol; HDL = High density lipoprotein; LDL = Low density lipoprotein; ALQ = Activities of lifestyle questionnaire; IADL = instrumental activities of daily living; MOSS = Medical Outcome Study Social Support Survey; and sd = standard deviation

### Disability status among CF and Non-CF participants after five years of follow-up

In this study, participants with CF more commonly reported manifestations of disability. CF participants reported more than double the percentage of experiencing disability at 24.1% as compared to 10.3% of the non-CF participants. Furthermore, Table 2 shows that the adjusted OR of reporting disability was 1.761 times greater (95% CI: 0.907, 3.422) among CF participants compared to those without CF (*p* < 0.05).

**Table 2:** Adjusted independent odd ratio of having disability after five years follow-up in participants with CF and comorbidity at baseline

Health condition	Disability		
	Adj OR	95% CI	*p*-value
CF	1.761	0.907, 3.422	0.045*
Hypertension	1.032	0.551, 1.931	0.922
Hypercholesterolemia	0.949	0.486, 1.854	0.877
Diabetes mellitus	1.536	0.267, 5.075	0.009*
Osteoarthritis	4.413	1.082, 17.992	0.038*
Heart diseases	1.844	0.692, 4.909	0.021*
Chronic kidney disease	2.876	0.185, 44.694	0.450
Cancer	2.572	0.235, 28.109	0.439
Depression	1.866	0.981, 3.546	0.043*
Age	2.498	1.169, 5.338	0.018*
Ethnicity	1.879	0.842, 4.196	0.124
Education years	1.296	0.552, 3.043	0.551
Household income	1.000	0.999, 1.000	0.058
Fasting blood glucose	1.069	0.921, 1.240	0.380
Skeletal muscle mass	1.006	0.918, 1.102	0.904
Body fat percentage	1.041	0.995, 1.089	0.083
Social support	0.965	0.943, 0.988	0.003*

* Significant at *p* < 0.05 using binary logistic regression after adjusting for age, ethnicity, education years, household income, fasting blood glucose, skeletal muscle mass, body fat percentage, and MOSS. Adj OR = Adjusted odd ratio; CI = Confidence interval; MCI; Mild cognitive impairment; CF = Cognitive frailty

### Independent associations between health conditions and incidence of disability

As shown in Table 2, the chronic health conditions included namely osteoarthritis (OR: 4.413, 95% CI: 1.082, 17.992), depression (OR: 1.866, 95% CI: 0.981, 3.546), heart diseases (OR: 1.844, 95% CI: 0.692, 4.909), and diabetes mellitus (OR: 1.536, 95% CI: 0.267, 5.075), were independently associated with increased adjusted OR of disability. Notably, osteoarthritis was associated with the highest independent OR of disability after adjusting for covariates (*p* < 0.05).

### Combined associations and synergistic effects between CF and comorbidities on disability

Table 3 indicates that the combined effects of CF and all significant comorbidities are linked to a heightened risk of disability, surpassing the independent effects of each condition. In particular, a synergistic effect was observed between CF and osteoarthritis, with participants reporting both conditions being 6.675 times more likely to report disability (95% CI: 1.057, 42.158) compared to those without either condition. The RERI between the two conditions was 1.501 (95% CI: 1.400, 1.570), suggesting that the combined effects of CF and osteoarthritis on disability are greater than the sum of their independent effects. Moreover, 22.5% of the total OR was attributable to this interaction (AP: 0.225, 95% CI: 0.160, 0.290).

**Table 3:** Adjusted odd ratios and synergistic effect measures of CF and comorbidity with regard to the likelihood of mild/moderate disability and severe disability

Variables	OR (95% CI)	*p*-value	RERI	AP	SI
CF + Hypertension	1.107 (0.539, 2.273)	0.782	-0.686 (-0.715, -0.657)	-0.619 (-0.648, -0.590)	0.135 (0.106, 0.164)
CF + Hypercholesterolemia	1.024 (0.444, 2.361)	0.956	-0.686 (-0.717, -0.555)	-0.670 (-0.701, -0.639)	0.033 (0.002, 0.064)
CF + Diabetes mellitus	2.904 (1.487, 5.671) *	0.002	0.607 (0.577, 0.637)	0.209 (0.178, 0.239)	1.468 (1.440, 1.528)
CF + Osteoarthritis	6.675 (1.057, 42.158) *	0.044	1.501 (1.400, 1.570)	0.225 (0.160, 0.290)	1.360 (1.300, 1.420)
CF + Heart diseases	3.480 (1.378, 8.786) *	0.041	0.875 (0.831, 0.919)	0.251 (0.207, 0.295)	1.545 (1.500, 1.590)
CF + CKD	1.681 (0.038, 12.246)	0.794	-2.884 (-3.010, -2.750)	-1.716 (-1.850, -1.590)	0.191 (0.061, 0.321)
CF + Cancer	3.117 (0.254, 38.229)	0.374	-1.144 (-1.260, -1.030)	-0.367 (-0.479, -0.255)	0.649 (0.537, 0.761)
CF + Depression	3.443 (1.065, 11.126)*	0.039	0.806 (0.753, 0.859)	0.234 (0.181, 0.287)	1.502 (1.450, 1.550)
Age	2.863 (1.355, 6.046)*	0.006			
Ethnicity	1.895 (0.877, 4.093)	0.104			
Education years	1.168 (0.502, 2.715)	0.718			
Household income	1.000 (0.999, 1.000)*	0.026			
Fasting blood glucose	1.068 (0.928, 1.229)	0.361			
Skeletal muscle mass	1.017 (0.928, 1.114)	0.721			
Body fat percentage	1.055 (1.008, 1.105)*	0.022			
Social support	0.969 (0.947, 0.991)*	0.007			

* Significant at *p* < 0.05 using binary logistic regression test after adjusting for age, ethnicity, education years, household income, fasting blood glucose, skeletal muscle mass, body fat percentage, and MOSS. * Notes: CF = cognitive frailty; CKD = Chronic kidney disease. Adjusted for age, ethnicity, education years, household income, fasting blood glucose, skeletal muscle mass, body fat percentage, and MOSS.

The synergistic effects between CF and heart diseases were also associated with an increased risk of disability incidence by 3.480 times greater (95% CI: 1.378, 8.786) than participants who did not report having either condition. The RERI was 0.875 (95% CI: 0.831, 0.919), also indicating that the synergistic effects of CF and heart diseases on disability are greater than the sum of the independent effects of each condition. Notably, 25% of the OR was attributable to this synergism (AP: 0.251, 95% CI: 0.207, 0.295).

The combined effects of CF and depression also increased the risk of disability by 3.443 (95% CI: 1.065, 11.126) times greater compared to those who did not report experiencing either condition. The RERI between the two conditions was 0.806 (95% CI: 0.753, 0.859), indicating that the synergistic effects of CF and depression on disability are greater than the sum of the independent effects of each condition. Of the total OR, 23.4% was attributable to this interaction (AP: 0.234, 95% CI: 0.181, 0.287). There were no synergistic effects between CF and other included comorbidity, including hypertension, hypercholesterolemia, chronic kidney disease, and cancer on the incidence of disability after five years follow up.

Synergistic effects were also observed between CF and diabetes mellitus, impacting the incidence of disability by 2.904 times greater (95% CI: 1.487, 5.671). The RERI was 0.607 (95% CI: 0.577, 0.637), indicating that the synergistic effects of CF and diabetes mellitus are greater than the sum of the independent effects of each condition. About 21% of the total OR was attributable to this synergism (AP: 0.209, 95% CI: 0.178, 0.239).

In the analysis, moderate multicollinearity was observed among the predictor variables, as indicated by the tolerance values ranging from 0.526 to 0.990 and corresponding VIF between 1.010 and 1.899. While these values suggest the presence of moderate collinearity, they remain below commonly accepted thresholds for severe multicollinearity, ensuring the reliability of the regression results.

## Discussion

Generally, the findings of this study indicated that the combined effect of CF and comorbidities on the incidence of disability exceeds the sum of their separate effects. In scenarios where resources are limited, it may be most practical to focus on subgroups where the greatest impact on disability prevention or intervention is expected by targeting CF. Hence, methods for assessing additive interaction using SI, AP, and RERI [36] can help to achieve this objective. In the previous longitudinal study, the synergistic effects of depression and comorbidity were associated with increased disability and decreased quality of life among older adults in Singapore by three-fold [37]. Additionally, both frailty and cognitive impairment were reported to have a synergistic effect with comorbidity on disability occurrence; however, these two syndromes were studied independently [38, 39]. To date, this is the first study of its kind to investigate the synergistic effects between CF and comorbidity in relation to disability incidence through a cohort prospective study among community-dwelling older adults in Malaysia.

The combined effect of CF and osteoarthritis on disability incidence was observed to be the highest by six times more as compared to those who reported either one of the conditions. Both conditions share important common risk factors and have also been established as predictors of falls and disability in older adults [15, 40]. Older adults with osteoarthritis-related pain are more prone to have decreased confidence in their balance [41], delayed walking speeds and poorer handgrip values [42], heightened psychological concern related to falls, and are less likely to be physically active [41], which consequently exacerbates physical impairment leading to a downwards trajectory into disability. Furthermore, osteoarthritis and CF are often accompanied by depression and lower physical performance as related pain may restrict daily activities and heighten the risk of depressive symptoms [13, 14, 41]. Tai Chi, a traditional Chinese practice involving slow, coordinated movements, mindfulness, and controlled breathing, demonstrates potential in enhancing physical and psychological health in individuals with knee osteoarthritis [43, 44] and MCI [45]. Moreover, emerging evidence suggests the effectiveness of multidomain interventions in mitigating the risks and reversing cognitive frailty (CF) and comorbidity in older adults [46, 47]. Therefore, integrating exercises with cognitive stimulation, nutritional support, and psychosocial interventions holds promise in reducing disability risk associated with comorbidities while enhancing cognitive function and emotional well-being.

This study also demonstrated the synergistic effect of older adults with CF and heart diseases on the incidence of disability by more than three-fold, as compared to those without both conditions. Heart diseases have been identified as the most common self-reported cause of the overall decline in functional status [48], which in turn progresses to disability in later life. Besides, research has demonstrated that reduced cerebral blood flow resulting from decreased cardiac output in individuals with heart failure can potentially lead to both sarcopenia and cognitive impairment [49, 50]. Furthermore, modifiable risk factors such as educational level, exercise capacity, sleep disturbance, and depressive symptoms are associated with an elevated likelihood of cognitive decline among individuals with heart failure, which can be addressed through non-pharmacological interventions [51, 52]. It has also been demonstrated that a combined program of aerobic exercise and cognitive training significantly improved verbal memory, self-care management, quality of life, and functional capacity in persons with heart failure [51, 53]. Thus, these findings highlights the potential effectiveness of multifaceted non-pharmacological interventions improving various aspects of well-being, with the potential to act as preventive measures against disability in later life.

Next, this study also reported that CF and depression had synergistic effects on disability incidence by three-fold greater than those who did not report either condition. A recent systematic review and meta-analysis indicated that the prevalence of CF with depression in older adults is high wherein both are mutually affected and share common physiologic processes (e.g. inflammation) and risk factors (e.g. physical inactivity) [54, 55]. Therefore, a possible pathological mechanism underlying these associations could be due to high inflammation levels, such as elevated interleukin-6 (IL-6), which impacts future adverse health problems, including the incidence of disability among older adults [56, 57]. Depressive symptoms have been identified as a significant predictor of CF incidence in previous research [13, 14], highlighting the importance of addressing depression in older individuals. Implementing interventions targeting depressive symptoms is crucial not only for mental health but also as a preventive measure against disability. Non-pharmacological interventions like psychotherapy, cognitive-behavioral therapy, and psychosocial support programs can be instrumental in treating depression among older adults [58], aiming to alleviate symptoms and enhance overall well-being, potentially reducing the risk of cognitive frailty and associated disabilities.

Among the older adults reported to have CF, diabetes mellitus demonstrated synergistic relationships with the incidence of disability. The combined effect of these two conditions is three-fold higher than these risk factors on its own, highlighting the importance of addressing the medical comorbidities in CF interventions. This is consistent with a published report that cognitive impairment and physical frailty are powerful prognostic factors in predicting disability and mortality among older adults with diabetes mellitus [59]. Metabolic and vascular dysregulation, characterized by hyperglycemia, dyslipidemia, and chronic inflammation, have been identified as the primary biological mechanisms underlying the observed synergistic relationship between diabetes and CF [60, 61]. Additionally, hypoglycemia in individuals with diabetes is linked to CF, depressive symptoms, low psychological well-being, and reduced quality of life. These factors can impede the performance of daily activities and increase the risk of disability [19]. Hence, prioritizing mental well-being through personalized care plans that include counseling, support groups, and fostering community connections is essential for improving overall resilience and quality of life among older adults confronting both cognitive frailty and diabetes mellitus.

To the best of our knowledge, this study is the first of its kind which specifically looks into the synergistic effects of individual comorbidities that co-occur with CF in Malaysian older adults on disability indices. After accounting for a broad range of confounding factors, the results of this longitudinal study shed light on the intricate and dynamic cause-and-effect relationship between the synergistic effects of CF and comorbidities on disability incidence among older adults in Malaysia. While the independent effects of CF and chronic diseases are expected, our findings are important as they highlight the multiplicative effects of co-existing CF and medical comorbidities on disability incidence. This study is not without its limitations. Firstly, data regarding the presence of comorbidities were self-reported which may be influenced by misunderstanding or inaccurate responses from the participants. However, it should be noted that self-reported disease diagnoses have been used widely and reported to be valid [29]. Secondly, it is important to note that we did not consider the interval between the initial diagnosis of comorbidities and the index date, as older adults with CF may not accurately provide this information. However, this data could have been obtained from the medical records of older adults, with prior instructions for them to bring along their medical follow-up records if available. Thirdly, the generalizability of the current findings to the general population may not be possible owing to the smaller sample size included in this analysis. Nonetheless, this study provides novel findings and can be a stepping stone for more in-depth, explorative future research undertakings. Hence, there is a need for future large-scale longitudinal studies with extended follow-up periods among older adults to validate our current findings. In future studies with a larger sample size, CF could be defined into several subtypes, and data could be stratified based on age, sex, educational levels, and regions to underscore the significance of cognitive frailty. Additionally, it is recommended that future research should prioritize interventional randomized controlled trials targeting both CF and comorbidities simultaneously as a proactive strategy to prevent, delay, or manage poor health outcomes among older adults.

## Conclusions

In conclusion, the findings of our study highlights the synergistic effects of CF and comorbidities, such as OA, HD, depressive symptoms, and diabetes mellitus, heightening the risk of disability in later life. Early identification of individuals at risk for both CF and comorbidities is crucial for preventing or mitigating disability in older populations. In addition, implementing interdisciplinary interventions targeting CF and associated comorbidities can delay disability onset, emphasizing the importance of integrated care models and community-based support systems to enhance the well-being of older adults.

## Data Availability

The datasets generated and/or analysed during the current study are not publicly available to protect the confidentiality and anonymity of study participants but are available from the corresponding author at reasonable request.
